# Supplementary material for: Efficacy of duloxetine compared with opioid for postoperative pain control following total knee arthroplasty
Source: PLoS One. 2021 Jul 2;16(7):e0253641. doi: 10.1371/journal.pone.0253641 (PMC8253394; doi:10.1371/journal.pone.0253641)
Supplement: Attachment — Submitted filename: Response to Reviewers.docx [file pone.0253641.s002.docx]

Dear authors,
Here you receive my review concerning the manuscript entitled “Efficacy of Duloxetine compared with opioid for postoperative pain control following total knee arthroplasty” for PLOS one (PONE-D-21-04527R1). The authors from the orthopedic surgery department present the results from their retrospective non-randomized single center observational study for the second time in their revised manuscript.

▶We thank the reviewer for his/her valuable time, and we agree with this succinct summary of our study.

Overall the authors took our previous comment very seriously. This improved the general understanding of the presented study, results and conclusion. However, I have a few remarks.

Ad Abstract:
Line 39 please add period at the end of the sentence.

▶Thank you for your comments. We added period at the end of the sentence in the revised manuscript. (Line 39)

Lines 42-43 …considered an alternative to opioid… . Sure, I may understand this conclusion or suggestion. But please consider patients allergic or intolerant for morphine or opioids. As you show in this small group that there were no significant benefits regarding , i.e., nausea and vomiting.

▶Thank you for your comments. We fully agree that there may be side effects from using opioid or duloxetine. More high quality large-scale randomized controlled trials are still required to further confirm the side effects of duloxetine. We added this issue in the abstract and conclusion of revised manuscript. (Lines 41-42 and 319-320)

Line 53 please change indiscreet into indiscrete

▶Thank you for your comments. We changed indiscreet into indiscrete in the revised manuscript. (Line 52)

Line 55 please change post-operative into postoperative

▶Thank you for your comments. We changed post-operative into postoperative in the revised manuscript. (Line 54)

Line 58 Here the abbreviation “OA” appears for the first time. Please write for the first time completely. You may mean osteoarthrosis or osteoarthritis?

▶Thank you for your comments. We added the full term of OA in the revised manuscript. (Line 57)

Patients and treatment data line 104: here the authors mention the number of 944 eligible patients, whereas in the abstract (Methods)the total number mentioned initially is 857 patients. Please explain, rewrite, or adapt?

▶Thank you for your comments. There was a mistake in abstract. The contents of Abstract have been modified in the revised manuscript. (Line 27)

Line 102: As mentioned before in our previous review duloxetine 30 mg is considered a low dose. The authors answered that during the 6 weeks duloxetine dose was increased to 60 mg. This should be mentioned in Methods and/or results. For instance by a statement or observation that after X weeks X% of patients took a total of 60 mg of duloxetine daily.

▶Thank you for your comments. In general, the dosage of duloxetine starts with 30 mg initially. It is increased to 60 mg, because this drug might have side effects. However, in our study, only duloxetine 30mg was used without dose increment, referring to previous studies that proved sufficient effect even with duloxetine 30mg. We added this issue in the method of the revised manuscript. (Lines 103-104)

Discussion Line326 please change the uppercase letter to lowercase regarding “Duloxetine”.

▶Thank you for your comments. We changed the uppercase letter to lowercase in the discussion of the revised manuscript. (Line 269)
